# ANGPTL4 Suppresses Clear Cell Renal Cell Carcinoma via Inhibition of Lysosomal Acid Lipase

**DOI:** 10.1158/2767-9764.CRC-24-0016

**Published:** 2024-08-27

**Authors:** Zeng Jin, Umasankar De, Tanzia Islam Tithi, Jeremy Kleberg, Akhila Nataraj, Elena Jolley, Madison E. Carelock, Brandon S. Davies, Weizhou Zhang, Ryan Kolb

**Affiliations:** 1 Cancer Biology Concentration, Biomedical Graduate Program, College of Medicine, University of Florida, Gainesville, Florida.; 2 Department of Pathology, Immunology and Laboratory Medicine, College of Medicine, University of Florida, Gainesville, Florida.; 3 Department of Biochemistry and Molecular Biology, College of Liberal Arts and Sciences, University of Florida, Gainesville, Florida.; 4 Department of Health Science, College of Public Health and Health Professions, University of Florida, Gainesville, Florida.; 5 Interdisciplinary Medical Sciences Division, Florida State University, Tallahassee, Florida.; 6 Department of Biochemistry and Molecular Biology, Fraternal Order of Eagles Diabetes Research Center, and Obesity Research and Education Initiative, University of Iowa, Iowa City, Iowa.; 7 UF Health Cancer Center, University of Florida, Gainesville, Florida.

## Abstract

Renal cell carcinoma (RCC), the most common form of kidney cancer, is a heterogeneous disease with clear cell RCC (ccRCC) being the most prevalent and aggressive subtype. While most ccRCC tumors have elevated expression of *angiopoietin-like*4 (*ANGPTL4*), in our study we identified a significant subset of patients whose cancers show no increase in *ANGPTL4* expression. These patients have a worse prognosis compared to the patients with high expression of *ANGPTL4*. These *ANGPTL4*-low cancers are characterized by the increased frequency of wild-type *Von Hippel-Lindau*(WT *VHL*), a gene that is commonly mutated in ccRCC, and an enrichment for genes associated with lipid metabolism. Using RCC tumor models with WT VHL, we demonstrate that ANGPTL4 behaves as a tumor suppressor. The loss of ANGPTL4 in ccRCC cell lines results in increased tumor growth and colony formation in a lysosomal acid lipase (LAL)-dependent manner, a phenotype rescued by the expression of N-terminus ANGPTL4. At the mechanistic level, the loss of ANGPTL4 increases LAL activity in ccRCC cells. These data suggest that ANGPTL4 enacts its tumor-suppressive effects in ccRCC by regulating LAL activity. Importantly, the identified patient cohort with low *ANGPTL4* expression may exhibit increased reliance on lipid metabolism, which can be a point of target for future therapy.

**Significance:**

Our data indicate angiopoietin-like 4 (ANGPTL4) acts as a tumor suppressor in clear cell renal cell carcinoma via regulating lipid metabolism and identifies a cohort of patients with lower expression of ANGPTL4 that are correlated with shorter survival.

## Introduction

Renal cell carcinomas (RCC) account for about 76,000 new cancer cases annually in the U.S. and 403,000 cases worldwide (1, 2). Clear cell RCC (ccRCC) is the most common and aggressive subtype, accounting for 75% to 85% of cases with a 5-year survival rate of 50% to 69% for all cases and only 10% for metastatic disease (3). ccRCC is characterized by the accumulation of lipid droplets in cancer cells giving them the characteristic “clear” cytoplasm under histological observation (2). The increased intracellular lipid contents are associated with the loss of the *Von Hippel-Lindau* (*VHL*) gene that occurs in about 80% of the patients due to somatic mutation or promoter hypermethylation (4). The absence of the VHL gene leads to the stabilization of hypoxia-inducing factors (HIF), resulting in the upregulation of HIF target genes including those involved in angiogenesis, lipid synthesis, and lipid uptake (5, 6). The first line of therapy for metastatic ccRCC is small molecule tyrosine kinase inhibitors (TKI) targeting angiogenic signaling pathways in combination with immune checkpoint inhibitors (ICIs; refs. 7–9). However, while this combination has shown some clinical efficacy, clinical trials have shown limited complete response rates ranging from 3% to 9% (10–13). This highlights the need to identify patients who are likely to respond to these treatments as well as those who are not.

One target HIF proteins is angiopoietin-like 4 (ANGPTL4; ref. 14). ANGPTL4 is a secretory multifunctional protein which is cleaved into two functional peptides—nANGPTL4 and cANGPTL4. The N-terminus coil-coiled domain is well-known for its role as an inhibitor of lipoprotein lipase (LPL). The C-terminus fibrinogen domain is involved in wound healing, angiogenesis, and vessel permeability (15, 16). The role of ANGPTL4 in cancer progression depends on the cancer type. In gastric cancer, the knockdown of ANGPTL4 suppresses cancer development, whereas, in breast cancer, contrasting results have been reported with either pro- or anti-tumorigenic effects (17–20). In ccRCC, studies have shown that ANGPTL4 is highly upregulated in most tumors but the role of ANGPTL4 has not been established (21, 22).

In our study, we report that while ANGPTL4 is highly upregulated in ccRCC compared to other types of cancers and normal kidney tissues, there is a cohort of 15% of patients in whom *ANGPTL4* is not elevated, and these patients without elevated *ANGPTL4* had a worse prognosis than those with high ANGPTL4. Using several ccRCC tumor models, we demonstrate a tumor-suppressive role of ANGPTL4 in a subset of RCC cells mainly due to its novel function in regulating lysosomal acid lipase (LAL) within cancer cells.

## Materials and Methods

### Cell lines, CRISPR-Cas9, stable transfection, and cell culture

Renca, HK-2, and CAKi-1 cells were purchased from ATCC. RCC4 empty vector cells were purchased from MilliporeSigma. 786O cells were obtained from Synthego. For CRISPR-Cas9–mediated knockout of ANGPTL4, CAKi-1 cells were sent to Synthego. 786O and CAKi-1 ANGPTL4 knockout pools were generated by Synthego using direct transfection of Caspase 9 protein and single guide RNA. Gene editing was verified and pooled cells were sent to us. Single clones were grown from pooled cells and *ANGPTL4* RNA was determined. Clones with knockout of *ANGPTL4* were combined to generate ANGPTL4 KO cells (A4KO). Clones with no knockout of ANGPTL4 for 786O were combined to generate 786O WT cells. No CAKi-1 clones without loss of ANGPTL4 were found so parental CAKi-1 cells were used as the WT control. RCC4 A4KO cells were generated by direct transfection with Caspase 9 protein and single guide RNA (Synthego) in the lab. Clones with ANGPTL4 knockout were identified and pooled as for CAKi-1 and 786O cells. To generate *LIPA*/*ANGPTL4* double KO cells, CAKi-1 A4KO cells were transfected with LentiCrispr V2 plasmid (a gift from Feng Zhang; Addgene plasmid #52961; http://n2t.net/addgene:52961; RRID: Addgene_52961; ref. 23) containing one of 3 guide RNAs; guide 1: GAG​TGA​AAT​TAT​CTCTTA​CT, guide 2: AGT​GAA​ATT​ATC​TCT​TAC​TG, or guide 3: TTA​CTG​GGG​ATT​CCC​TAG​TG. Transfected cells were selected with puromycin. KO of *LIPA* in pooled cells was verified by Western blot for LAL. For the generation of Renca-A4 cells, cells were transfected with pHS2 plasmid containing V5-tagged human ANGPTL4 (24) and stably transfected cells were selected with Blasticidin. All cells were cultured in DMEM supplemented with 10% fetal bovine serum (FBS) plus penicillin/streptomycin. For siRNA transfection, cells were transfected with ON-TARGETplus set of 4 siRNA against *LIPA* or control non-targeting siRNA (Dharmacon) using GeneTran TM III transfection reagent (Biomiga) as per standard protocols provided by Dharmacon. The knockdown of *LIPA* was determined by Western blot for LAL. siRNA sequences are CGU​UUG​CAC​UCA​UGU​CAU​A, UGU​CUA​GAG​UGG​AUG​UAU​A, CAA​AUU​AGG​ACG​AUU​ACC​A, and GAA​CCA​UUC​UGA​CAA​AGG​U.

### *In vivo* tumor models

All animal studies were approved by the Institutional Animal Care and Use Committee at the University of Florida. Cells were suspended in 50/50 PBS/Matrigel (Corning NY). A total of 2 × 10^6^ or 5 × 10^6^ 786O and CAKi-1 WT or A4KO cells in 300 μL were injected subcutaneously into the flank of nod-scid IL2 receptor gamma^null^ (NSG) mice obtained from Jackson Laboratories (Bar Harbor ME). A total of 1 × 10^4^ Renca or Renca-A4 cells in 100 μL were injected into the flank of Balb/C mice (Charles Rivers Laboratories). Tumor growth was monitored by palpation with calipers and volume was determined as L × W^2^ × 0.5. For mice treated with antibodies, mice were treated with either 200 μg isotype control rat IgG (BioXcell) or anti-cANGPTL4 antibody clone 6A11A7 (20) every 4 days once tumors were palpable.

### Antibody purification

Anti-cANGPTL4 clone 6A11A7 hybridoma cells (20) were grown in rolling bottle in RPMI media supplemented with 10% FBS for about 14 days until approximately 70% cell death. Supernatant was collected by vacuum filtration. Antibodies were purified using gravity chromatography with protein G agarose beads (ThermoFisher Scientific). Antibodies were eluted with 3 mol/L glycine pH 3.0 and dialyzed against PBS. Antibodies were concentrated using 50 K molecular weight cut off spin filters (MilliporeSigma). Antibody purity and specificity were verified by SDS-page gel electrophoresis and ability to detect recombinant cANGPTL4 (RnD Systems) by Western blot, respectively.

### Flow cytometry

Tumor samples were digested to single cells using collagenase IV. Cells were suspended in PBS and stained with the following antibodies diluted 1:100: CD45 clone 30F11, CD34 clone HM34, CD140a clone APA5 (eBioscience), CD140b clone APB5, CD146 clone P1H12, CD90.2 clone 30-H12, VEGFR2 clone Avas12, CD31 clone 390, PDL1 clone 10F.9G2, CD62P clone RMP-1, and ICAM-1 clone YN1/1.7.4. Cells were also stained with fixable viability dye 780 (eBioscience). All antibodies were from Biolegend unless specified otherwise. All flow cytometry were ran using a Cytek Aurora 3 flow cytometer (Cytek Biosciences) and analyzed using FloJo software (BD Biosciences).

### Measurement of free fatty acids and cholesterol

The concentration of free fatty acids and total cholesterol were determined using the free fatty acid quantification kit (MilliporeSigma) and the cholesterol quantification kit (MilliporeSigma), respectively.

### Lysosomal acid activity assay

Lysosomal acid activity was measured using the LysoLive LAL assay kit (Abcam). The LysoLive probe was dissolved in DMSO as per the manufacturer’s instructions, then diluted 1:6,000 in serum-free DMEM. Cells were incubated with the diluted probe for 4 hours at 37°C. For the rescue experiments, A4KO cells were mock transfected, transfected with ANGPTL4-V5 (24) or transfected with truncated ANGPTL4-V5 (1–160) containing just the N-terminus (24). Cells were then suspended in the provided flow holding and sorting buffer and analyzed by flow cytometry.

### Colony formation

#### Nonadherent colony growth

A total of 4 × 10^4^ CAKi-1 WT and A4KO cells, 4 × 10^4^ RCC4 WT and A4KO cells, 1 × 10^4^ 786O WT and A4KO cells were seeded in 24-well ultra-low adherence plates in DMEM supplemented with 10% FBS plus penicillin/streptomycin and incubated for 72 hours. For experiments with the anti-cANGPTL4 antibody, 1 mg/mL of antibody or rat IgG was added to the culture media. For experiments with Lalistat 1, 10 μmol/L Lalistat 1 dissolved in DMSO or DMSO was added to the culture media. For the rescue experiments, A4KO cells were mock transfected, transfected with ANGPTL4-V5 (24) or transfected with truncated ANGPTL4-V5 (1–160) containing just the N-terminus (24). For siRNA experiments, cells were transfected with siRNA targeting *LIPA* or nontargeting control siRNA for 24 hours prior to plating on ultra-low adherence plates. The number of cells per well were counted using an inverted light microscope.

#### 0.5% methylcellulose colony growth

A total of 4 × 10^4^ CAKi-1 WT and A4KO cells, 4 × 10^4^ RCC4 WT and A4KO cells, 1 × 10^4^ 786O WT and A4KO cells were seeded in 24-well ultra-low adherence plates in DMEM supplemented with 10% FBS plus penicillin/streptomycin. 2% methylcellulose solution was added to a final concentration of 0.5% methylcellulose. Cells were incubated for 72 hours and the number of colonies per well were counted using an inverted light microscope.

#### Soft agar colony growth

A 0.5% solution of agar in complete media was added to six-well plates and allowed to solidify at room temperature. A total of 4,000 cells were suspended in 2× complete media and diluted 1:1 with 0.5% soft agar solution to obtain 0.25% agar. Cells were then plated on the solidified agar in the plates. Cells were incubated at 37°C for 30 days being fed with fresh complete media every 3 days. The number of colonies was counted using an inverted light microscope.

### MTS and CSFE proliferation assays

A total of 1,000 cells of CAKI-1 or 786O with both WT or ANGPTL4 KO were seeded in the 96-well plate with 200 μl of medium. MTS reagent powder (Promega) was prepared with a concentration of 2 mg/mL. Phenazine methosulfate (Sigma-Aldrich) was prepared with a concentration of 1 mg/mL. MTS and PMS was mixed with a ratio of 20:1 before adding to each individual wells. After addition of the MTS-PMS mixture, the plate was incubated for 4 hours at 37°C, and the absorbance was read at 490 nm wavelength.

For the CSFE experiment, 1 × 10^6^ of 786O cells were stained with 5 μmol/L of CellTrace CFSE (ThermoFisher Scientific) for 20 minutes at room temperature. The cells were washed and resuspended in fresh prewarmed complete culture medium. A total of 1 × 10^5^ of 786O cells were seeded in each well in a six-well plate. The plate was incubated for 24 hours and the CFSE signal was detected using Cytek Aurora flow cytometer.

### RNA isolation and real-time qPCR

RNA was isolated from cells using the NucleoSpin RNA mini kit (Macherey-Nagel). cDNA was generated from RNA using the SuperScript II First-Strand Synthesis kit (ThermoFisher Scientific). Quantitative real-time PCR was done using Power Up Sybr Green master mix (ThermoFisher Scientific). Primers for *ANGPTL4* were 5′-TAG​TCC​ACT​CTG​CCT​CTC​CC-3′ and 5′-GAG​ATG​GCC​CAG​CCA​GTT-3′. Primers for *GAPDH* were 5′- GGA​GCG​AGA​TCC​CTC​CAA​AAT -3′ and 5′- GGC​TGT​TGT​CAT​ACT​TCT​CAT​GG -3′.

### Immunohistochemistry

Formalin-fixed, paraffin embedded tumor sections were deparaffinized by sequential washes in xylene, 100% ethanol, 95% ethanol, and 70% ethanol. Slides were incubated at 95°C for 45 minutes in citrate antigen retrieval buffer (Abcam). Peroxidase activity blocked with 3% H_2_O_2_ and slides were blocked with background punisher (Biocare Medical). Cells were then incubated with anti-CD31 clone JC/70A (ThermoFisher Scientific) diluted 1:100 in Da Vinci green diluent (Biocare Medical). Slides were washed and incubated with mouse-on-mouse HRP polymer (Biocare Medical) for 20 minutes. Staining was developed with the betazoid 3,3′-diaminobenzidine chromagen kit (Biocare Medical). Slides were then counterstained with hematoxylin and mounted. Images were taken with a EVOS M5000 imaging system (ThermoFisher Scientific) and analyzed using ImageJ software.

### Cell migration

Cell migration was determined by transwell migration assay as per standard protocols. Briefly, 1 × 10^4^ cells were plated on the inner membrane of the transwell insert. Cells were then placed in the six-well plate with complete media. After 16 hours, cells were cleaned from the upper surface of the inner membrane and cells were stained. Cells that migrated to the outer surface of the membrane were counted.

### Western blot

Cells were lysed with RIPA buffer containing 150 mmol/L NaCl, 50 mmol/L Tris-HCL, 1% NP40, 0.5% sodium deoxycholate, 0.1% SDS, 1 mmol/L dithiothreitol, 2 μg/mL aprotinin, 1 μg/mL pepstatin A, 10 μg/mL leupeptin, 1 mmol/L PMSF. Proteins were separated by SDS-PAGE gel electrophoresis and visualized by Western blot. Antibodies used were anti-V5 Tag monoclonal antibody clone SV5-Pk1 (ThermoFisher Scientific) and anti-α-tubulin clone B-5-1-2 (MiliporeSigma), anti-GAPDH clone D16H11 (CellSignaling), and anti-LAL catalog 54155 (Novus Biologicals).

### RNA sequencing and transcriptome analysis

*ANGPTL4* pan cancer expression was determined using the pan cancer normalized The Cancer Genome Atlas program (TCGA) data. Graph was generated by the Human Protein Atlas (proteinatlas.org; ref. 25). Normal tissue expression data were obtained from genotype-tissue expression project and compared with TCGA data. The pathway analysis was performed using data of patients with ccRCC from TCGA comparing low and high *ANGPTL4* expression. *ANGPTL4* expression and patient survival TCGA KIRC (ccRCC) data were obtained using the Xena browser (xenabrowser.net; ref. 26). A robust *Z*-score was calculated for *ANGPTL4* and a distribution curve was generated using GraphPad Prism software (GraphPad Software). A robust *Z*-score of ≤−3.5 was considered to be low expression of *ANGPTL4* and the rest of the samples were considered bulk samples. A Kaplan–Meier survival curve was generated by separating samples into low *ANGPTL4* and bulk samples. A robust *Z*-score of ≤−1.5 was used because there were not enough samples in the ≤−3.5 group with non-censured survival data (follow up of more than 100 days) to obtain significance. Gene Set Enrichment Analysis (GSEA; refs. 27, 28) was performed compared *ANGPTL4*-low to bulk samples. Select gene sets related to lipid metabolism, angiogenesis, and immunology were identified, which had a false discovery rate (FDR) of <0.25. TCGA Illumina 450 K array methylation data for probes associated with *VHL* was obtained using the Xena browser. Correlation between probe methylation and *VHL* expression was determined with probes with Pearson R <−0.35 was considered to have a significant negative correlation. Methylation of two probes was identified to be negatively correlated with *VHL* expression and the % methylation of these two probes in the *ANGPTL4*-low and bulk samples was determined.

For RNAseq, RNA from WT and A4KO cells was purified and sent to the University of Florida Interdisciplinary Center for Biotechnology Research Gene Expression and Genotyping Core (RRID: SCR_019145). RNA concentration was determined by Qubit and quality was determined using a Bioanalyzer RNA assay. Samples with a RIN score ≥ 8 were used for library preparation. Library preparation was done using the NEBNext Poly(A) mRNA isolation module (New England Biolabs) and NEBNext Ultra II Library prep Kit (New England Biolabs). Libraries were sequenced at 150 paired end reads at 3 million reads/sample using an Illumina Novaseq 6000 (Illumina). Raw FastQ sequencing data were aligned to the hg38 genome using Kallisto pseudoalignment (29). Differential gene expression analysis was conducted using limma package (30). Pathway analysis was performed using GSEA software (27, 28). Graphs were generated using ggplot2 package in R.

### Annexin V PI staining

Annexin V propidium iodide (PI) staining was done using the FITS-Annexin V apoptosis detection kit (BD Biosciences). Cells were plated on six-well plates in triplicate and cultured in DMEM, 10% FBS, and pen/strep for 48 hours. Cells were trypsinized, washed two times, and suspended in 1× annexin V binding buffer and incubated with FITC-Annexin V and PI staining solution. Cells were then analyzed using an Aurora Cytek 3 spectral cytometer. Percent apoptosis was determined as the number of FITC-positive cells as a percentage of single cells.

### Statistical analysis

For comparison between two groups, Welch’s *t* test was done to determine the significance. For comparisons between three and more groups, a one-way ANOVA with Dunnett’s test to correct for multiple comparisons was done to determine significance. For survival data, log-rank test was done to determine significance. For tumor growth and cell growth curves a two-way ANOVA was done to determine significance. For transcriptomic analysis, *P* values were corrected using the Benjamini–Hochberg method. A *P* value of ≤0.5 was considered significant. For GSEA, a FDR of ≤0.25 was considered significant. Unless specified otherwise, all graphs were generated and statistical analysis done using GraphPad Prism software.

### Data availability

All RNAseq data were submitted to Gene Expression Omnibus datasets and can be accessed under GSE273236.

## Results

### A subset of patients with ccRCC with significantly lower ANGPTL4 is associated with shorter survival and lipid metabolism

**Figure 1:**
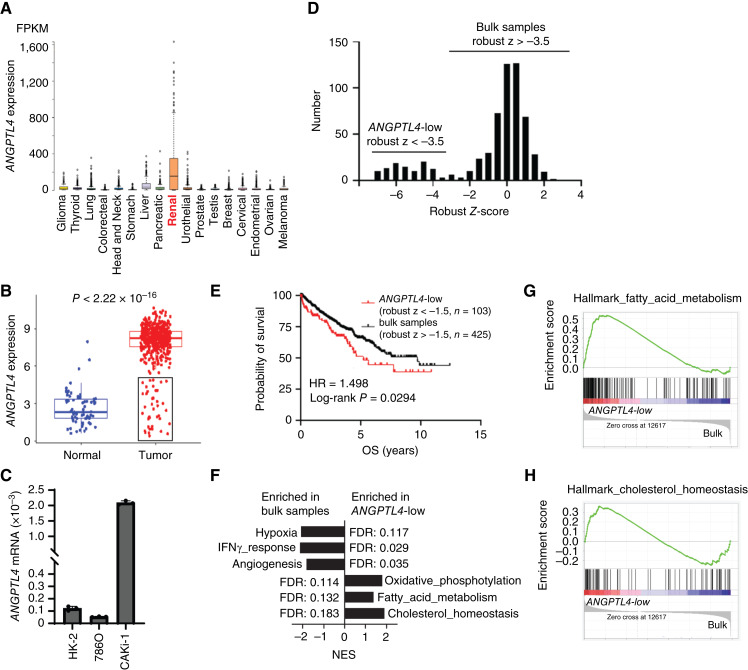
Subset of patients with ccRCC with low expression of ANGPTL4 is associated with shorter survival and lipid metabolism. **A,***ANGPTL4* expression in the indicated cancer types. Data generated from TCGA pan-cancer normalized RNAseq data generated using the Human Protein Atlas. **B,***ANGPTL4* mRNA expression in TCGA ccRCC samples compared to normal kidney tissue samples. **C,***ANGPTL4* expression in the indicated human ccRCC cell lines or immortalized human proximal tubule cells (HK-2) relative to *GAPDH*. **D,** Distribution of *ANGPTL4* mRNA levels in TCGA ccRCC (KIRC) samples. Graph depicts the number of samples within the indicated robust *Z*-score range. A subset of patients with significantly lower *ANGPTL4* expression (robust *Z*-score ≤ −3.5) is indicated as *ANGPTL4*-low distinguishable from the rest of the samples labeled as bulk samples. **E,** Kaplan–Meier curve depicting overall survival (OS) of ccRCC samples with low expression of ANGPTL4 compared to the rest of the sample. **F,** GSEA of TCGA samples comparing *ANGPTL4*-low (robust *Z* ≤ −3.5) and bulk samples (robust *Z* > −3.5). Graph depicts the nominal enrichment score (NES) of indicated gene sets enriched in the indicated group. The FDR is indicated. **G** and **H,** Enrichment plot for the indicated gene set from the GSEA in **F**.

The first line of therapy for metastatic ccRCC is antiangiogenic TKIs combined with immune checkpoint inhibitors. However, only a subset of patients shows a complete response and most develop resistance to the TKIs (10, 31). We looked at alternative angiogenic factors in ccRCC that can potentially be targeted in combination with immunotherapy. Analysis of TCGA pan cancer data showed that renal cancer has the highest expression of *ANGPTL4* compared to 16 other solid cancers (Fig. 1A). ccRCC tumors also had significantly higher expression of *ANGPTL4* compared to normal kidney tissue (Fig. 1B). Analysis of human ccRCC cell lines and an immortalized human proximal tubule cell line, HK-2, found varying relative expression of *ANGPTL4*, with similar expression in 786O and HK-2 cells, with 786O expression being slightly lower, and the highest expression in CAKi-1 cells (Fig. 1C). These data collaborate with previous studies that have shown high ANGPTL4 levels in ccRCC (21, 22). Gene Ontology pathway analysis shows that high expression of *ANGPTL4* is associated with hypoxia-induced factor 1 (HIF1) and peroxisome proliferator-activated receptor (PPAR) pathways (Supplementary Fig. S1A), which is consistent with previous studies demonstrating both pathways can lead to the production of ANGPTL4 (14, 32).

Interestingly, a closer examination of the expression data indicated a subset of patients with ccRCC without elevated expression of *ANGPTL4* (Fig. 1B—black box). Further analysis of *ANGPTL4* expression in ccRCC found that a subset of patients (approximately 15%) had significantly lower expression of *ANGPTL4* compared to most ccRCC tumors (Fig. 1D, robust *Z*-score of <−3.5). Separating the samples into *ANGPTL4*-low and bulk samples, we found that the *ANGPTL4*-low group had shorter overall survival [hazard ratio (HR) = 1.498, log-rank; *P* = 0.0294] compared to the rest of the samples (bulk samples; Fig. 1E). Here we used a robust *Z*-score of <−1.5, as there were not enough samples in the group with a robust *Z*-score of <−3.5 with survival data for statistical analysis. This indicates that while most patients with ccRCC have elevated expression of *ANGPTL4*, the subset of patients with ccRCC without elevated expression of *ANGPTL4* may have a worse prognosis.

Loss of VHL function results in the upregulation of HIFs—a driving factor of ccRCC. As ANGPTL4 is regulated by HIF1, we examined *VHL* mutation status in the ANGPTL4-low group compared to the bulk samples. Of the samples with mutation data, only 2/22 (9%) had mutant VHL in the ANGPTL4-low group compared to 161/310 (52%) for the bulk samples (Supplementary Fig. S1B). VHL loss of function in ccRCC can also be due to promoter hypermethylation (4, 33). We examined VHL promoter methylation in the ANGPTL4-low group and bulk samples and identified two probes from the TCGA Illumina 450 K methylation data where methylation was negatively correlated with VHL expression (Supplementary Fig. S1C and S1D). The ANGPTL4-low group had significantly lower methylation of these two probes compared to the bulk samples (Supplementary Fig. S1E and S1F). This suggests that the expression of ANGPTL4 in human ccRCC tumors is regulated in part by VHL status. It is important to note, however, that CAKi-1 cells, which have wild-type VHL, have the highest expression of *ANGPTL4* (Fig. 1C) among all ccRCC cell lines, indicating that there are other regulatory mechanisms for *ANGPTL4* expression in ccRCC.

To better understand how this subset of patients with low ANGPTL4 differs from the bulk patients we performed GSEA (27, 28). The bulk samples were enriched for gene sets related to HIF signaling such as hypoxia and angiogenesis as well as immune-related gene sets such as interferon-gamma (IFNγ; Fig. 1F). These gene sets are related to the current therapy paradigm of targeting angiogenesis in combination with immunotherapy. In contrast, the ANGPTL4-low group showed enrichment for gene sets related to lipid metabolism including fatty acid metabolism and cholesterol metabolism (Fig. 1F–H), suggesting that this subgroup of tumors may be driven by lipid metabolism.

### ANGPTL4 suppresses tumor growth of ccRCC cells with WT VHL

**Figure 2:**
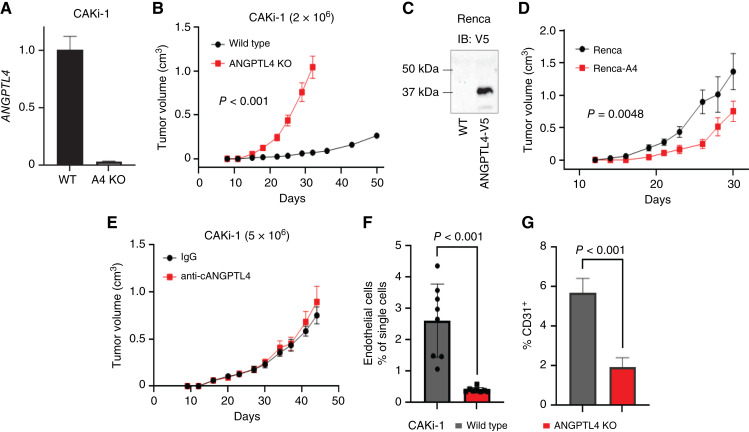
ANGPTL4 suppresses tumor growth of RCC cells with wild-type VHL. **A,** Graph depicts expression of *ANGPTL4* in CAKi-1 WT or A4KO cells relative to *PPIA* as a fold change compared to WT. **B,** 2 × 10^6^ of the indicated CAKi-1 cells were implanted into the flank of NOD-SCID/IL2RG (NSG) mice. Graph depicts the average tumor volume ± SEM. Two-way ANOVA was done to determine significance (*n* = 8 for both groups). **C,** Renca mouse RCC cells were stably selected for expression of human ANGPTL4-V5. Immunoblot for V5 in the indicated Renca cells. **D,** Renca or Renca cells expressing human ANGPTL4-V5 (Renca-A4) from (**C**) were implanted into the mammary gland of Balb/C mice. Graph depicts the average tumor volume ± SEM. Two-way ANOVA was done to determine significance (*n* = 8 WT, *n* = 7 Renca-A4). **E,** 5 × 10^6^ of CAKi-1 cells were implanted into the flank of NSG mice. CAKi-1 tumor–bearing mice were treated with 200 μg anti-cANGPTL4 antibody or rat IgG isotype control twice weekly starting at day 14. Graph depicts the average tumor volume ± SEM. **F,** Graph depicts the number of CD31^+^/CD34^+^ endothelial cells ± SD as a % of single cells from the indicated tumor from (**B**). Welch’s *t* test was done to determine significance. **G,** Tumor sections from the indicated tumors in **B,** were stained for CD31. Graph depicts the average CD31^+^ staining as a % of total area ± SD. At least three images from three samples were used for each group. Welch’s *t* test was done to determine significance.

To understand the effect of ANGPTL4 on tumor progression, we used CRISPR-Cas9 to generate KO of ANGPTL4 in CAKi-1 cells, a human ccRCC cell line that has WT VHL. Individual clones were selected with KO of ANGPTL4 (Supplementary Fig. S2A) and combined to generate the CAKi-1 ANGPTL4 KO cell line (referred to as CAKi-1 A4KO, Fig. 2A). We also used the same method to generate ANGPTL4 KO versions of 786O and RCC4 cells, human ccRCC cell lines with mutant VHL (Supplementary Fig. S2B and S2C). As shown in Fig. 2B, loss of ANGPTL4 led to significantly increased CAKi-1 tumor growth in immunocompromised mice (Fig. 2B). To further confirm the tumor growth suppressive function of ANGPTL4, we generated Renca mouse renal cell carcinoma cells with WT VHL, which express V5-tagged ANGPTL4 (Fig. 2C, Renca-A4). Renca-A4 cells had significantly decreased tumor growth compared to control Renca cells (Fig. 2D), further supporting the tumor suppressive function of ANGPTL4 in RCC. ANGPTL4 is a secreted protein that is cleaved into two peptides with different functions, nANGPTL4 and cANGPTL4 (34). To further understand which ANGPTL4 signaling peptide suppresses tumor growth, we treated the CAKi-1 tumor–bearing mice with a cANGPTL4 blocking antibody, which we have previously shown inhibits cANGPTL4-mediated angiogenesis both *in vitro* and *in vivo* (20). Blocking cANGPTL4 had no effect on tumor growth (Fig. 2E). Similar results were found for both Renca (Supplementary Fig. S2D) and Renca-A4 (Supplementary Fig. S2E) tumors. These data suggest that the tumor suppressive effect is not mediated via secreted cANGPTL4 but via nANGPTL4. As cANGPTL4 is known to promote angiogenesis, we next checked to see if loss of ANGPTL4 affects the tumor vasculature. Loss of ANGPTL4 resulted in a decrease in the number of CD31^+^ endothelial cells, indicating reduced tumor vascularization (Fig. 2F and G; Supplementary Fig. S2F). Further suggesting that the tumor-suppressive function of ANGPTL4 is via the N-terminus function and is independent of the proangiogenic function of the c-terminus. However, this tumor suppressive function of ANGPTL4 is not observed in all ccRCC cells. Knockout of ANGPTL4 in 786O cells which have mutant *VHL*, significantly reduced tumor growth (Supplementary Fig. S2G) as did treatment with anti-cANGPTL4 antibodies (Supplementary Fig. S2H), suggesting that in 786O cells, the proangiogenic tumor-promoting function of cANGPTL4 may be predominant over the tumor suppressive function.

### ANGPTL4 suppresses ccRCC colony formation

**Figure 3:**
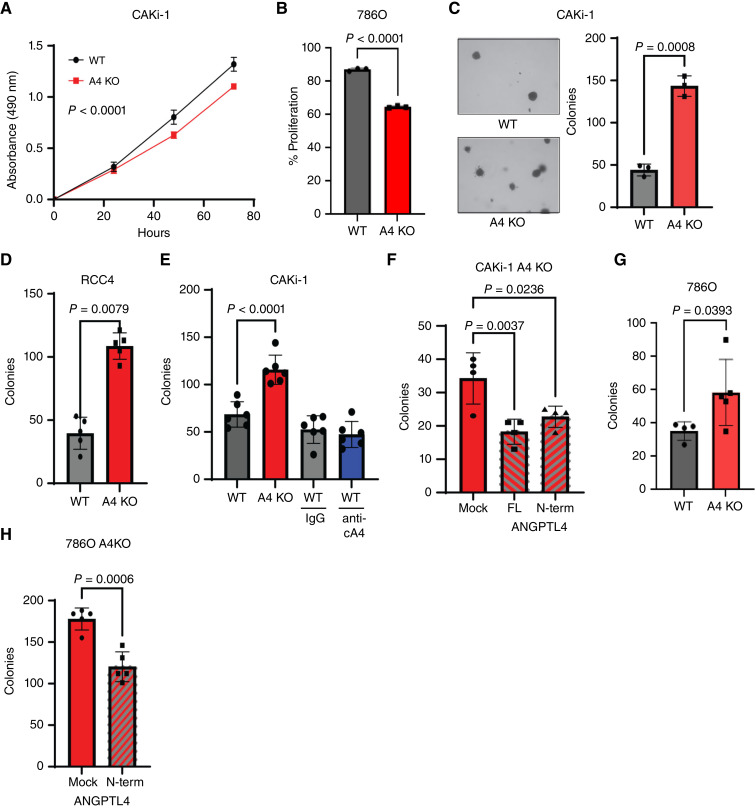
nANGPTL4 suppresses ccRCC colony formation. **A,** MTS assay of the indicated CAKi-1 cells. Graph depicts the average absorbance ± SD. Two-way ANOVA was done to determine significance (*n* = 6 all groups). **B,** The indicated 786O cells were incubated with CSFE and analyzed by flow cytometry. The graph depicts the average % proliferation of the cells ± SD. Welch’s *t* test was done to determine significance. **C,** Soft agar colony assay. The indicated CAKi-1 cells were cultured on soft agar for 30 days and the number of colonies per well was counted. Left: representative images of colonies from the indicated cells (10× magnification). Right: graph depicts the average number of colonies ± SD. Welch’s *t* test was used to determine significance. **D,** The indicated RCC4 cell line was incubated in nonadherent conditions for 72 hours. The graph depicts the average number of colonies/well ± SD. Welch’s *t* test was done to determine significance. **E,** Graph depicts the average number of colonies/well of the indicated CAKi-1 cells treated as indicated with rat IgG or anti-cANGPTL4 ± SD. One-way ANOVA with Dunnett’s test to correct for multiple comparisons was done to determine significance. **F,** Graph depicts the average number of colonies per well ± SD. of CAKi-1 A4 KO cells transfected with full length (FL), N-terminus or mock transfected as indicated. One-way ANOVA with Dunnett’s test to correct for multiple comparisons was done to determine significance. **G,** Graph depicts the average number of colonies/well of the indicated 786O cells ± SD. Welch’s *t* test was done to determine significance. **H,** Graph depicts the average number of colonies/well ± SD of 786O A4 KO cells transfected with N-terminus or mock transfected as indicated. One-way ANOVA with Dunnett’s test to correct for multiple comparisons was done to determine significance.

To confirm the functional role of ANGPTL4 in suppressing tumor growth, we performed a series of *in vitro* experiments on A4KO and WT ccRCC cells. *In vitro* proliferation assays indicated that loss of ANGPTL4 led to slightly decreased cell proliferation in CAKi-1 (Fig. 3A) and 786O (Fig. 3B; Supplementary Fig. S3A and S3B) cells. The loss of ANGPTL4 in CAKi-1 cells led to significantly reduced cell migration (Supplementary Fig. S3C), which is consistent with previous studies showing that ANGPTL4 can promote cancer cell migration (35, 36). As these results may suggest a tumor-promoting function for ANGPTL4, we next looked at the colony formation ability under different conditions. Loss of ANGPTL4 in CAKi-1 cells increased soft agar colony formation (Fig. 3C)–an assay of anchorage-independent cell growth and an indicator of the tumorigenic potential of cells. We next checked the ability of the cells to form colonies in nonadherent conditions which depends on the presence of self-renewing populations and resistance to anoikis (37). Loss of ANGPTL4 in both RCC4 and CAKi-1 cells significantly increased colony formation in non-adherent culture conditions. (Fig. 3D and E; Supplementary Fig. S3D). This phenotype was not recapitulated by treatment of CAKi-1 WT cells with an anti-cANGPTL4 (anti-cA4) blocking antibody (Fig. 3E). To verify the role of ANGPTL4 in suppressing colony formation and to determine which signaling peptide (cANGPTL4 or nANGPTL4) was responsible, we transfected CAKi-1 A4KO cells with either V5-tagged full length (FL) ANGPTL4 or nANGPTL4 (Supplementary Fig. S3E). Exogenous expression of full length ANGPTL4 or nANGPTL4 reduced colony formation in A4KO cells compared to mock transfected control cells (Fig. 3F). Similar results were obtained for 786O cells where the loss of ANGPTL4 increased colony formation (Fig. 3G) and exogenous expression of nANGPTL4 reversed the phenotype (Fig. 3H; Supplementary Fig. S3F). We also determined colony formation with 0.5% methylcellulose in the culture media, which helps protect cells when grown in suspension, and obtained similar results with increased colony formation in CAKi-1 A4KO cells compared to WT (Supplementary Fig. S3G). These data strongly indicate that nANGPTL4 suppresses ccRCC cell colony formation either by a cellular intrinsic mechanism or via autocrine signaling.

### Absence of ANGPTL4 upregulates genes associated with lipid metabolism

**Figure 4:**
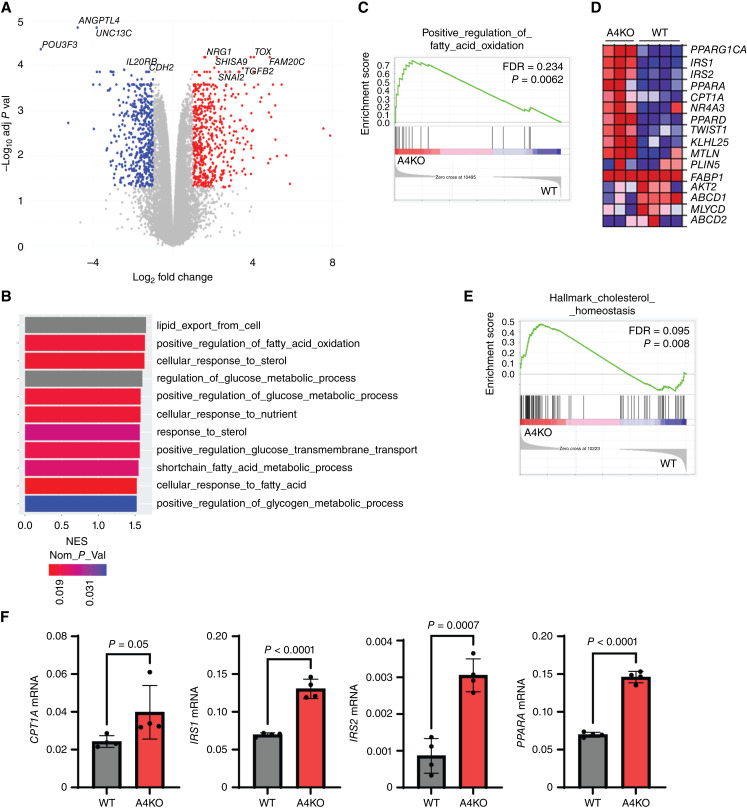
Loss of ANGPTL4 is associated with enrichment for lipid metabolism genes. **A,** Volcano plot for CAKi1 A4KO versus WT cells. Genes with an absolute value log_2_ fold change ≥1 and an adjusted *P* value of ≤0.05 are indicated. Genes upregulated in A4 KO cells are red and genes downregulated in A4KO cells are blue. **B,** GSEA was performed for CAKi-1 A4KO and WT cells. Graph depicts the nominal enrichment score (NES) of gene sets related to metabolism that were enriched—had a FDR <0.25—with A4KO cells. The nominal *P* value is indicated by the heat bar. Gray bars had a nominal *P* value that was lower than the threshold for the package used and thus had a value of 0. **C,** Enrichment plot for the indicated gene set from the GSEA analysis in **B**. The FDR and nominal *P* value are indicated. **D,** Heatmap of the genes in the gene set from **C,** for CAKi-1 A4KO and WT cells. **E,** GSEA analysis was performed for 786O A4KO and WT cells. Enrichment plot for the indicated gene set showing enrichment in A4KO cells. The FDR and nominal *P* value are indicated. **F,** Graphs depict the average mRNA expression relative to *PPIA* of the indicated genes in CAKi-1 WT and A4KO cells ± SD. Welch’s *t* test was done to determine significance.

To understand the molecular mechanism of ANGPTL4 in suppressing tumor growth, we performed RNA-seq analysis on the ccRCC cell lines with ANGPTL4 KO. There were 1,205 differentially regulated genes with a Log_2_ fold change of ≥ |1| and an adjusted *P* value of ≤0.05 in CAKi-1 A4KO cells compared to WT (Fig. 4A). We performed GSEA and found that loss of ANGPTL4 led to an enrichment of genes related to two major pathways: (i) immune related response, including several genesets related to chemotaxis of immune cells, and (ii) metabolism (Fig. 4B; Supplementary Fig. S4A). Interestingly, there is an enrichment in genes related to fatty acid beta-oxidation and the cellular response to cholesterol (Fig. 4B and C), suggesting that loss of ANGPTL4 may modify lipid metabolism. Several genes related to fatty acid metabolism are increased in CAKi-1 A4KO cells including *PPARGC1A*, *IRS1*, *IRS2*, and *CPT1A* (Fig. 4D). Similar results were found for 786O A4KO cells (Supplementary Fig. S4B). There was an enrichment for genes related to fatty acid oxidation (Supplementary Fig. S4C), however, it did not reach a FDR of <0.25, possibly due to the smaller sample size. Many of the same genes enriched in CAKi-1 A4KO cells were also enriched in 786O A4KO cells including *CPT1A*, *IRS2*, and *PPARGC1A* (Supplementary Fig. S4C, bottom). We also found an enrichment in genes related to cholesterol homeostasis in 786O A4KO cells (Fig. 4E). The differential expression of *CPT1A*, *IRS1*, *IRS2*, and *PPARA* in CAKi-1 A4KO cells compared to WT cells was verified by real-time PCR (Fig. 4F). As ANGPTL4 is known to regulate lipid metabolism, these data strongly suggest that ANGPTL4 may suppress ccRCC tumor progression via regulating lipid metabolism.

### ANGPTL4 suppresses ccRCC via regulation of LAL

**Figure 5:**
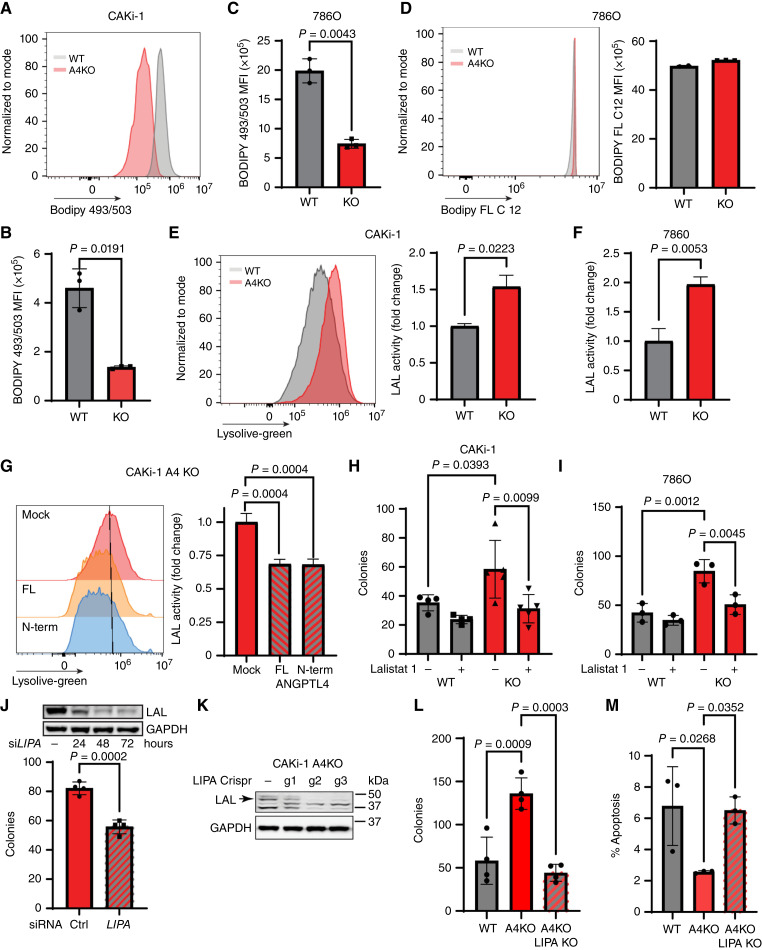
nANGPTL4 inhibits LAL to suppress ccRCC colony formation. **A–C,** Total intracellular lipids in the indicated CAKi-1 or 786O cells were stained with BODIPY 493/503 and the median fluorescence intensity (MFI) was determined. **A,** Representative histogram is presented for BODIPY staining of CAKi WT (gray) and A4KO (red) cells. **B** and **C,** Graph depicts the average BODIPY 493/503 MFI of the indicated CAKi-1 (**B**) and 786O (**C**) cells ± SD. Welch’s *t* test was used to determine significance. **D,** CAKi-1 WT and A4KO cells were incubated with BODIPY FL C12 and analyzed by flow cytometry to measure lipid uptake. Left: representative histogram showing BODIPY FL C12 fluorescent intensity of WT (gray) and A4KO (red) cells. Right: graph depicts the average BODIPY FL C12 MFI ± SD in the indicated 786O cells. Welch’s *t* test was done to determine significance. **E,** LAL activity was determined by incubating the indicated cells with lysolive-green and measuring the fluorescence Intensity by flow cytometry. Left: representative histogram depicting intensity of lysolive-green fluorescence in CAKi-1 WT (gray) and A4KO (red) cells. Right: graph depicts the average lysolive-green MFI as a fold change compared to WT ± SD. Welch’s *t* test was done to determine significance. **F,** Graph depicts the average lysolive-green MFI as a fold change compared to WT ± SD. Welch’s *t* test was done to determine significance. **G,** CAKi-1 A4KO cells were mock transfected or transfected with full length (FL) or N-terminus (N-term) ANGPTl4 and incubated with lysolive-green. Left representative histrogram depicting lysolive-green fluorescence intensity in the indicated cell lines. The peak fluorescence intensity in mock transfected cells is indicated by the black line. Right: Average lysolive-green MFI as a fold changed compared to mock transfected ± SD. One-way ANOVA with Dunnett’s test to correct for multiple comparisons was done to determine significance. **H,** Graph depicts the average number of colonies/well of the indicated CAKi-1 cells grown in the presence or absence of the LAL inhibitor lalistat 1. One-way ANOVA with Dunnett’s test to correct for multiple comparisons was done to determine significance. **I,** Graph depicts the average number of colonies/well of the indicated 786O cells grown in the presence or absence of the LAL inhibitor lalistat 1. One-way ANOVA with Dunnett’s test to correct for multiple comparisons was done to determine significance. **J,** CAKi-1 A4KO cells were transfected with siRNA targeting *LIPA* (gene that encodes LAL) or nontargeting control siRNA (−). Cells were plated in non-adherent conditions, cultured for 72 hours and the number of colonies was counted. Top: western blot for LAL at the indicated time point post transfection. GAPDH blot is used as a loading control. Bottom: graph depicts the average number of colonies per well ± SD. Welch’s *t* test was done to determine significance. **K,** CAKi-1 A4KO cells were transfected with a lentivirus plasmid containing CRISPR-Cas9 and one of the three guide RNAs targeting *LIPA*. After selection total lysates were collected and a Western blot for LAL and GAPDH, as a loading control, was done. Arrow indicates LAL band. **L,** The indicated CAKi-1 cells (A4KO LIPA KO cells are g2 cells from **K**) were cultured in nonadherent conditions for 72 hours and the number of colonies was counted. Graph depicts the number of colonies per well ± SD. One-way ANOVA with Dunnett’s test to correct for multiple comparisons was done to determine significance. **M,** The indicated CAKi-1 cells were cultured for 72 hours, stained with annexin V-FITC and PI and analyzed by flow cytometry. Graph depicts the % of FITC-positive cells as a percentage of single cells ± SD. One-way ANOVA with Dunnett’s test to correct for multiple comparisons was done to determine significance.

As we found that loss of ANGPTL4 led to the enrichment of genes related to lipid metabolism (Fig. 4), we next checked to see any differences between lipid storage and processing in A4KO cells compared to WT cells. We first looked at the amount of free fatty acids and total cholesterol in the cells. We found that while there was a decrease in free fatty acids in CAKi-1 A4KO cells (Supplementary Fig. S5A, left), there was no difference in 786O cells (Supplementary Fig. S5A, right). There was no difference in total cholesterol in either cell line (Supplementary Fig. S5B). Using BODIPY 493/503 to stain neutral lipids, we found that loss of ANGPTL4 resulted in a decrease in total lipid storage in both CAKi-1 cells (Fig. 5A and B) and 786O cells (Fig. 5C; Supplementary Fig. S5C). To determine if this decrease in lipids was due to a decrease in lipid uptake, we stained cells with BODIPY FL C12 and found a nonsignificant decrease in lipid uptake in CAKi-1 A4KO cells and no difference in 786O cells (Fig. 5D; Supplementary Fig. S5D). This suggests that the decrease in lipids in the A4KO cells is not due to a decrease in lipid uptake but rather possibly due to an increase in lipid utilization and/or export.

Our data suggest that the loss of nANGPTL4 is responsible for the suppressive phenotype observed in A4KO cells (Fig. 4). The known function of nANGPTL4 is the inhibition of LPL, however, this does not explain the decrease in lipids seen in our A4KO cells. If the phenotype was due to increased LPL activity, we would expect to see an increase in intracellular lipids as more LPL activity would increase the amount of lipids available for the cells to uptake. As such, we next looked at intracellular lipases. A previous study found that loss of LAL resulted in decreased colony formation and tumor growth of ccRCC cells (38), a phenotype that mirrors the one we observed in CAKi-1 A4KO cells (Figs. 2 and 3). As such, we used a fluorescent probe to measure LAL activity and found that loss of ANGPTL4 resulted in increased LAL activity in both CAKi-1 (Fig. 5E) and 786O (Fig. 5F; Supplementary Fig. S5E) cells. To verify that the increase in LAL activity was due to the loss of ANGPTL4, we exogenously expressed either FL ANGPTL4 or nANGPTL4 in CAKi-1 A4KO cells and measured LAL activity. Exogenous expression of both FL and N-terminus ANGPTL4 reduced LAL activity (Fig. 5G) indicating that LAL activity can be regulated by nANGPTL4. To determine if the increase in LAL activity seen in A4KO cells was responsible to the increase in colony formation, we treated CAKi-1 and 786O WT and A4KO cells with lalistat 1, a selective LAL inhibitor (39). Inhibition of LAL reduced colony formation of both CAKi-1 (Fig. 5H) and 786O (Fig. 5I) A4KO cells in nonadherent culture conditions. siRNA-mediated knockdown of *LIPA*, the gene that encodes LAL, (Fig. 5J; top) also reduced colony formation in nonadherent culture conditions. We next used the CRISPR-Cas9 system to generate *ANGPTL4*/*LIPA* double knockout CAKi-1 cells (Fig. 5K). As shown in Fig. 3, CAKi-1 A4KO cells had increased soft agar colony formation compared to WT cells. This phenotype was reversed with the knockout of *LIPA* (Fig. 5L). These data indicate that the tumor suppressive function of ANGPTL4 in ccRCC cells is due to inhibition of LAL activity by the N-terminus peptide. Interestingly, knockout of *LIPA* in CAKi-1 A4KO cells did not rescue the decreased lipid storage as there was reduced lipids in both CAKi-1 A4KO and CAKi-1 A4KO LIPA KO cells (Supplementary Fig. S5F), suggesting that the regulation of lipid storage by ANGPTL4 in CAKi-1 cells is not mediated by regulation of LAL. As previous studies have indicated that LAL promotes ccRCC cell survival (38), we next checked to see the effect of A4KO on cell survival. Knockout of ANGPTL4 in CAKi-1 cells increased cell survival compared to WT cells, a phenotype that was reversed by the knockout of *LIPA* (Fig. 5M; Supplementary Fig. S5G). These data suggest that ANGPTL4 may suppress tumor progression in CAKi-1 cells via inhibiting LAL-mediated survival mechanisms.

## Discussion

In our research, we discovered that the protein ANGPTL4 is notably overexpressed in ccRCC. However, we also identified a cohort of patients—approximately 15%—with significantly lower expression of ANGPTL4 and who have a less-favorable prognosis. Analysis of these patients found that they had a lower frequency of VHL mutations, indicating that the high levels of ANGPTL4 in ccRCC is at least partially due to the high percentage of tumors with inactive VHL. Tumors with low ANGPTL4 were enriched for genes related to lipid metabolism, while tumors with high ANGPTL4 were enriched for genes related to angiogenesis (Fig. 1). In our animal experiments, we established that ANGPTL4 significantly suppresses tumor growth in human CAKi-1 and mouse Renca RCC cells with WT VHL. The tumor growth acceleration does not appear to be influenced by the extrinsic function of cANGPTL4, as blocking antibodies failed to alter tumor growth (Fig. 2; Supplementary Fig. S2). Instead, knocking out ANGPTL4 leads to enhanced colony formation and reintroduction of either the full-length or the N-terminus portion of the protein can diminish the increase in colony formation (Fig. 3; Supplementary Fig. S3). Furthermore, ANGPTL4 KO cells display lower intracellular lipid levels coupled with higher LAL activity levels. Notably, inhibiting LAL as well as knockdown or knockout of LAL leads to a decrease in colony formation in the ANGPTL4 KO cells (Fig. 5; Supplementary Fig. S5). Suggesting that the regulation of LAL by nANGPTL4 is at least partially responsible for its tumor suppressive function.

Although these findings suggest that ANGPTL4 has an intrinsic role in inhibiting LAL to regulate lipid metabolism and suppress ccRCC tumor growth, there are still questions remaining, and several key directions and strategies can be considered. It is possible that the increase in LAL activity due to loss of ANGPTL4 allows cells to better utilize stored lipids for energy, to generate metabolic intermediaries and generate bioactive lipids and lipid-based signaling molecules. This has been shown in endothelial cells, where a recent publication found that knockdown of ANGPTL4 resulted in increased fatty acid oxidation and decreased glucose utilization (40). Previously it was shown that LAL promotes ccRCC survival and proliferation by increasing the generation of 14,15-epoxyeicosatrienoic acids—an arachidonic acid–derived signaling molecule (33). In support of a survival-mediated mechanism, we found a loss of ANGPTL4 in CAKi-1 cells decreases apoptosis, while this phenotype is reversed in ANGPTL4/LIPA double knockout cells (Fig. 5M). As this increase in survival was minor, there may be other mechanisms by which nANGPTL4 suppresses ccRCC. A deeper understanding of the molecular mechanisms underlying ANGPTL4’s intrinsic function in inhibiting LAL and the resulting changes to lipid metabolism and composition should be pursued.

This suppressive phenotype, however, is not present in all ccRCC cell lines, as knockout of ANGPTL4 or inhibition by treatment with anti-cANGPTL4 *inhibits* tumor growth in the 786O tumor model, a model with mutant VHL (Supplementary Fig. S2G and S2H). These data suggest that while ANGPTL4 can act as a tumor suppressor, likely via an intrinsic function of the n-terminus, some ccRCC cell lines are more dependent on the tumor-promoting proangiogenic function of cANGPTL4 for tumor progression. We hypothesize that the tumor suppressive function of ANGPTL4 plays a bigger role in the *ANGPTL4*-low subset of patients identified in Fig. 1, while tumors with high *ANGPTL4* may be more dependent on angiogenesis for tumor progression. This is supported by data showing that loss of ANGPTL4 in CAKi-1 tumors reduced angiogenesis, despite there being an increase in tumor growth. While in 786O cells, treatment with anti-cANGPTL4 antibodies which block cANGPTL4-mediated angiogenesis both *in vitro* and *in vivo*, (20) reduces tumor growth (Supplementary Fig. S2). The suppression of LAL activity and colony formation by nANGPTL4 was observed in both cell lines (Figs 3 and 5) indicating that the nANGPTL4 function is active in both cell lines. This hypothesis, while supported by our data, needs further evidence to support it. A better understanding of which tumors are more reliant on the tumor-promoting proangiogenic function of cANGPTL4 may be important for the future development of therapies for ccRCC.

Due to relatively low response rates to current frontline therapy for ccRCC—combining anti-angiogenic TKIs with immune checkpoint inhibitors (10, 13)—it is important to identify which patients are likely to respond as well as those that are not in order to provide the most effective therapy. Here we identified a cohort of patients with ccRCC with low expression of ANGPTL4 and a lower frequency of VHL mutations that are correlated with shorter overall survival (Fig. 1). These tumors were characterized with an enrichment for genes related to lipid metabolism (Fig. 1F–H), and our tumor models suggest that lipid regulation via LAL may be a particularly important driver of tumor progression in these tumors. While in the 786O tumor model, loss of ANGPTL4 or treatment with anti-cANGPTL4 antibodies had the opposite effect, reducing tumor growth. The implications of these findings may be that patients with low levels of ANGPTL4 may be less likely to respond to current therapy and need a different therapeutic approach. The inhibition of LAL as a potential strategy to reduce tumor growth could be used as a therapeutic development, however, the enzyme plays a critical role in regulating fatty acid metabolism as the KO of the gene results in severe dysregulation of energy homeostasis in mice (41) and humans with LAL mutations often suffer from lethality at a young age (42, 43). Therefore, targeting other enzymes involved in the fatty acid metabolism pathway to reduce this process can be a focus for developing therapeutics for this subset of patients.

In summary, we identified a cohort of patients with ccRCC with significantly lower expression of ANGPTL4 that are correlated with shorter survival. Further studies indicate that ANGPTL4 suppresses ccRCC tumor growth possibly via inhibiting cancer cell intrinsic LAL. The increase in LAL activity may alter the cells ability to utilize lipids for energy and signaling molecules to promote survival. Thus, this cohort of patients may be responsive to therapies targeting lipid metabolism pathways. Further studies to delineate the tumor suppressive function of nANGPTL4 and the tumor-promoting function of cANGPTL4 and ways to identify which pathway may be more important in which tumors is warranted.

## Supplementary Material

Supplementary Figure S5 Supplementary Figure S5

Supplementary Figure S1 Supplementary Figure S1

Supplementary Figure S2 Supplementary Figure S2

Supplementary Figure S3 Supplementary Figure S3

Supplementary Figure S4 Supplementary Figure S4
