# Supplementary material for: ANGPTL4 Suppresses Clear Cell Renal Cell Carcinoma via Inhibition of Lysosomal Acid Lipase
Source: Cancer Res Commun. 2024 Aug 27;4(8):2242–54. doi: 10.1158/2767-9764.CRC-24-0016 (PMC11348483; doi:10.1158/2767-9764.CRC-24-0016)
Supplement: Supplementary Figure S5 [file crc-24-0016_supplementary_figure_s5_suppsf5.docx]

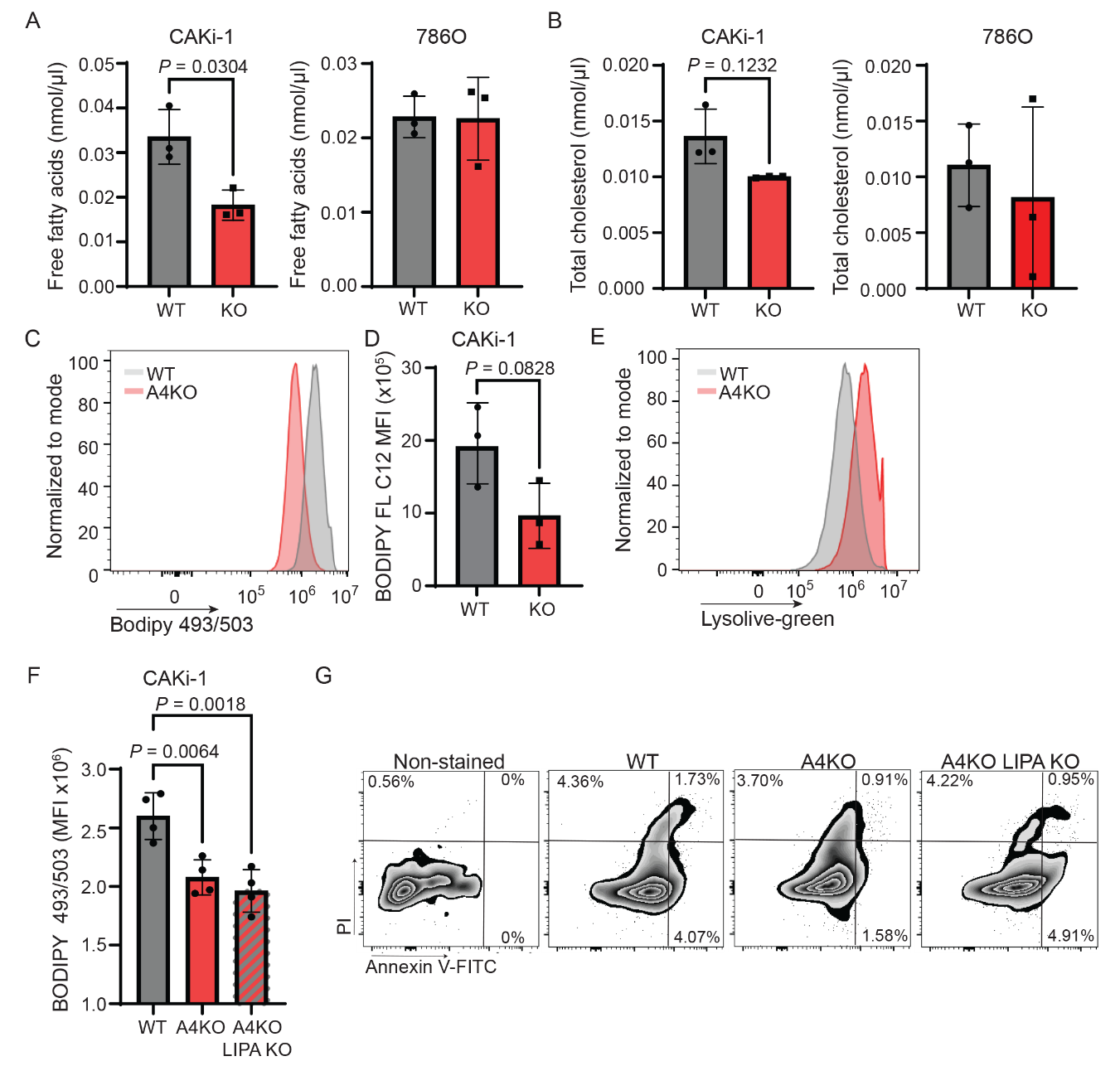


**Supplemental Figure S5.** A) Average concentration of free fatty acids in the indicated CAKi-1 (left panel) and 786O (right panel) ± s.d. Welch’s t test was done to determine significance. B) Average concentration of total cholesterol in the indicated CAKi-1 (left panel) and 786O (right panel) ± s.d. Welch’s t test was done to determine significance. C) Representative histogram of the indicated 786O cells incubated with BODIPY 493/503. D) CAKi-1 WT or A4KO (KO) cells were incubated with Bodipy FL C12 and analyzed by flow cytometry. Graph depicts lipid uptake as indicated by BODIPY FL C12 MFI ± s.d. Welch’s t test was done to determine significance. E) Representative histogram of LysoLive-green fluorescence in the indicated 786O cells. F) The indicated CAKi-1 cells were incubated with BODIPY 493/503 and analyzed by flow cytometry. The graph depicts the total lipid content as indicated by BODIPY 493/503 MFI ± s.d. One-way anova with Dunnett’s test to correct for multiple comparisons was done to determine significance. G) The indicated CAKi-1 cells were stained with annexin V-FITC and propidium iodide (PI) and analyzed by flow cytometry. Representative zebra plots showing gating for PI and FITC positive gating. Percentage indicates the percent of cells in the indicated gate compared to total single cells.
