# Supplementary material for: ANGPTL4 Suppresses Clear Cell Renal Cell Carcinoma via Inhibition of Lysosomal Acid Lipase
Source: Cancer Res Commun. 2024 Aug 27;4(8):2242–54. doi: 10.1158/2767-9764.CRC-24-0016 (PMC11348483; doi:10.1158/2767-9764.CRC-24-0016)
Supplement: Supplementary Figure S1 [file crc-24-0016_supplementary_figure_s1_suppsf1.docx]

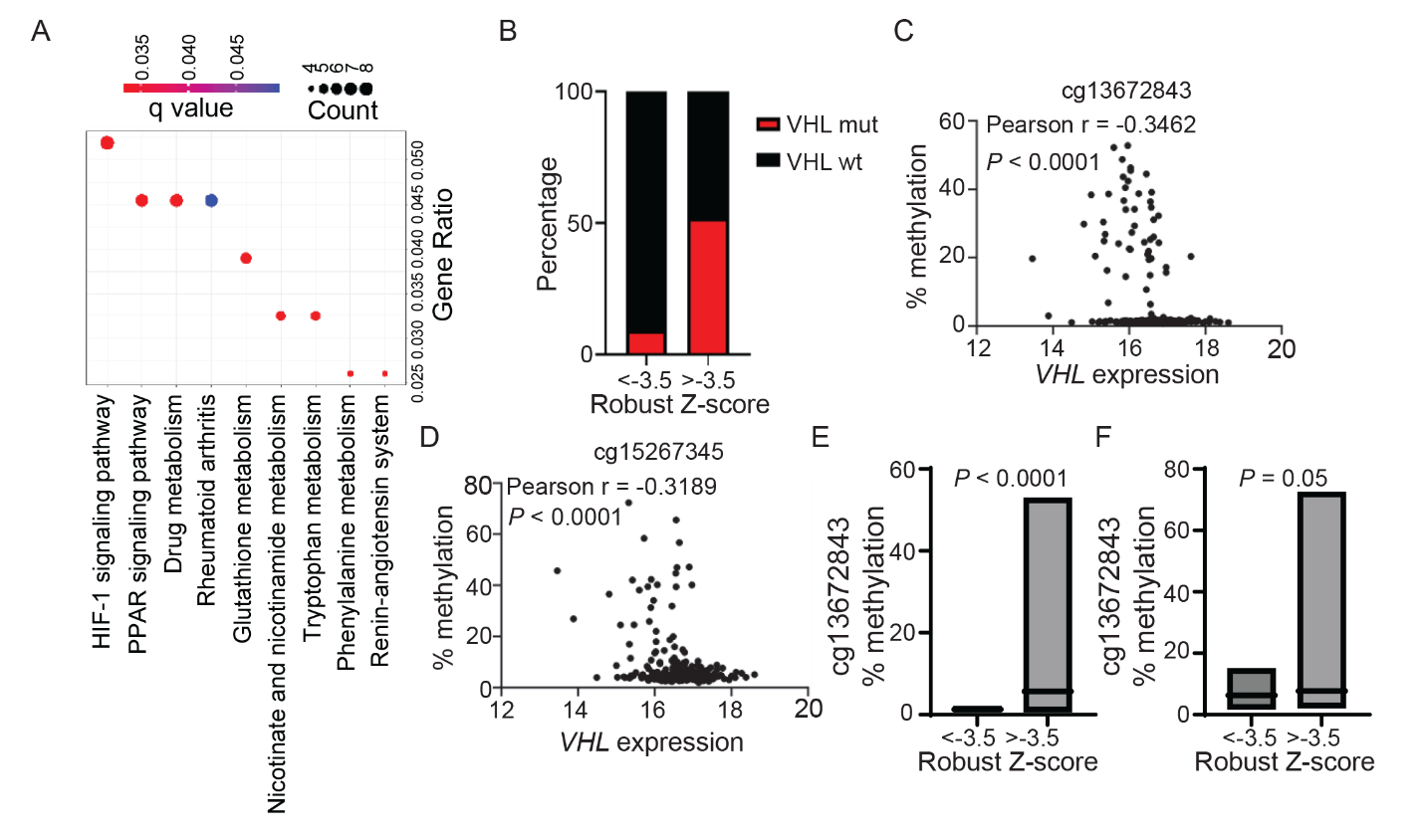


**Supplemental Figure S1.** A) GO pathway analysis of ccRCC samples from TCGA divided into ANGPTL4 high and low groups. Graph depicts the pathways associated with *ANGPTL4* expression. The size of the dot is correlated with the number of genes in the pathway that are differentially expressed in the *ANGPTL4* high group. Color corresponds to the Q value. B). Graph depicts the percentage of samples with wild type (wt) or mutant VHL in TCGA ccRCC samples with the indicated robust Z-score for *ANGPTL4*. C-D) correlation between *VHL* mutation and percent methylation of the indicated probe from the TCGA Illumina 450K methylation dataset. E-F) Graphs depict the % methylation of the indicated probe in ccRCC TCGA samples divided into groups with the indicated robust Z-score for *ANGPTL4*. Welch’s t test was done to determine significance.
