# Supplementary material for: ANGPTL4 Suppresses Clear Cell Renal Cell Carcinoma via Inhibition of Lysosomal Acid Lipase
Source: Cancer Res Commun. 2024 Aug 27;4(8):2242–54. doi: 10.1158/2767-9764.CRC-24-0016 (PMC11348483; doi:10.1158/2767-9764.CRC-24-0016)
Supplement: Supplementary Figure S2 [file crc-24-0016_supplementary_figure_s2_suppsf2.docx]

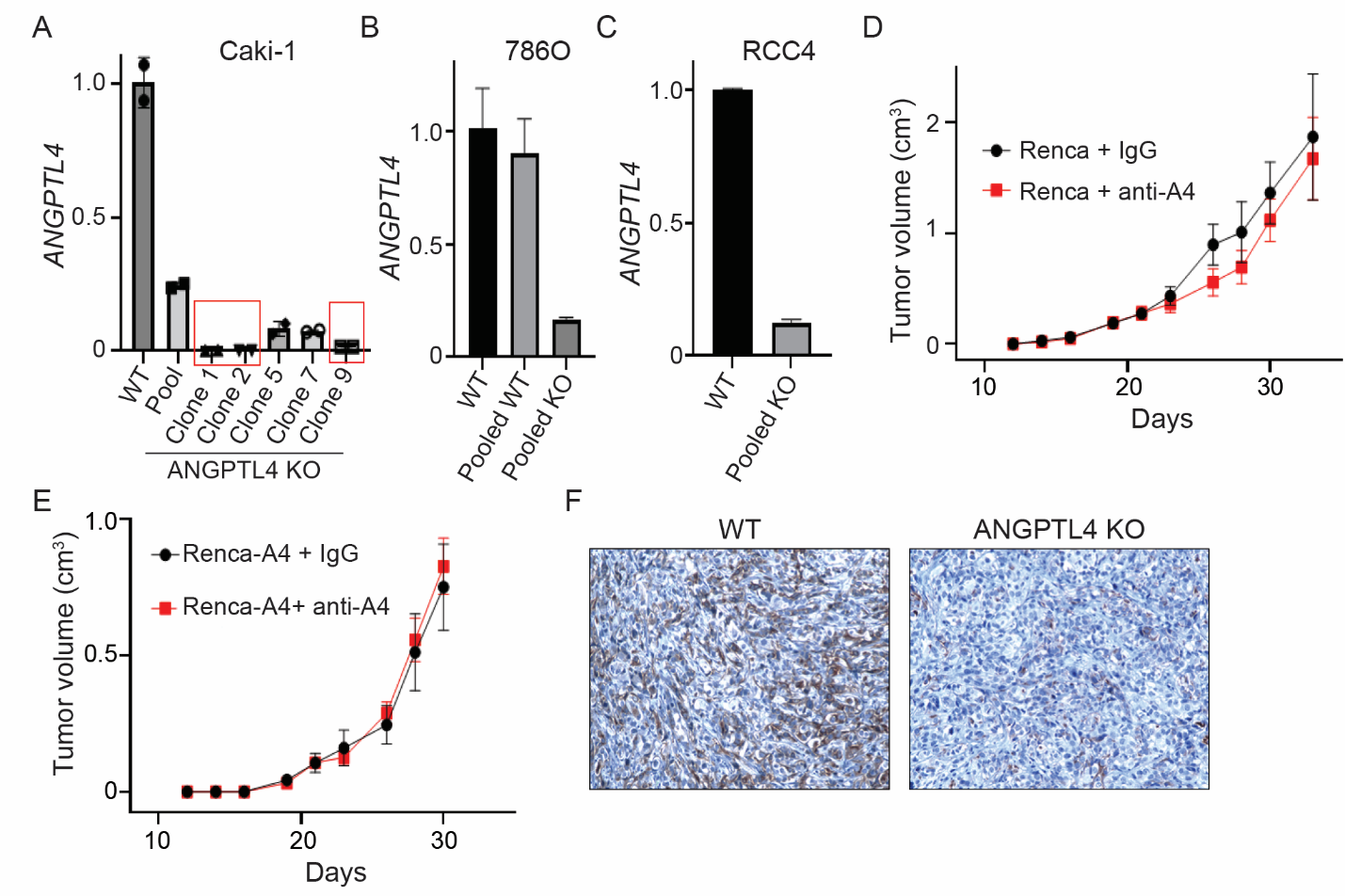
**Supplemental Figure S2.** A) individual clones were selected from pooled CAKI-1 *ANGPTL4* knockout (KO) cells generated using Crispr Cas9 system. Graph depicts *ANGPTL4* expression relative to *PPIA* as a fold change compared to wild type (WT) in the Indicated CAKi-1 cells. The clones in the red box were combined to create CAKi-1 A4KO cells. B) Individual 786O clones with or without Crispr Cas9 mediated ANGPTL4 KO were combined to generate pooled KO (referred to as 786O A4KO) and pooled WT cell lines, respectively. Graph depicts the average *ANGPTL4* expression relative to *PPIA* as a fold change compared to parental 7860 WT cells ± s.d. C) Individual RCC4 clones with Crispr Cas9 mediated ANGPTL4 KO were combined to generate pooled KO (referred to as RCC4 A4KO). Graph depicts the average *ANGPTL4* expression relative to *PPIA* as a fold change compared to parental RCC4 WT cells ± s.d. D-E) Renca (D) and Renca cells expressing human ANGPTL4 (Renca-A4, E) were implanted into Balb/C mice and treated with isotype control rat IgG (IgG) or cANGPTL4 blocking antibody (anti-A4). Graphs depict the average tumor volume ± s.e.m. (n=6 per group). F) Representative IHC staining for CD31 in sections from indicated CAKi-1 tumors from Figure 2B.
