# Supplementary material for: ANGPTL4 Suppresses Clear Cell Renal Cell Carcinoma via Inhibition of Lysosomal Acid Lipase
Source: Cancer Res Commun. 2024 Aug 27;4(8):2242–54. doi: 10.1158/2767-9764.CRC-24-0016 (PMC11348483; doi:10.1158/2767-9764.CRC-24-0016)
Supplement: Supplementary Figure S3 [file crc-24-0016_supplementary_figure_s3_suppsf3.docx]

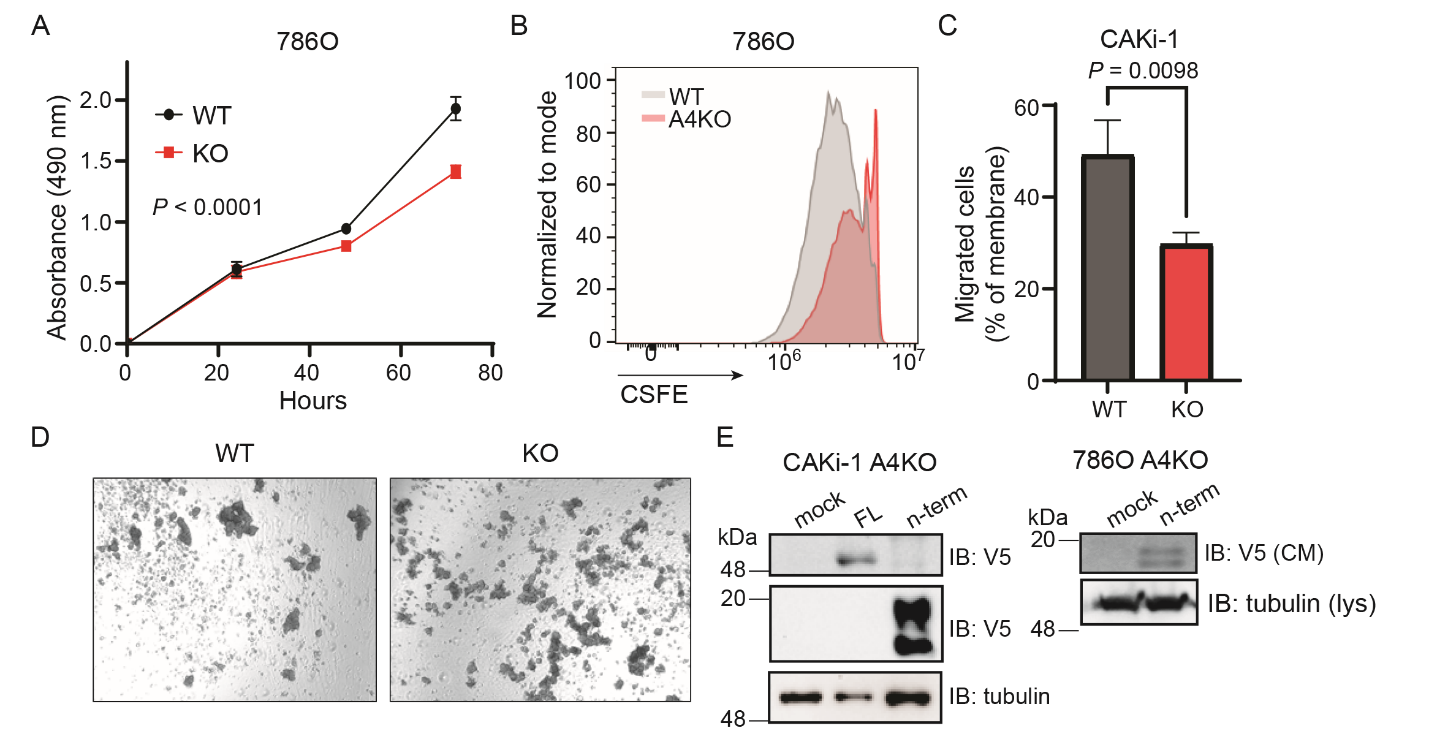


**Supplemental Figure S3.** A) MTS assay with 786O WT or A4KO cells. Graph depicts the average absorbance ± s.d. A two-way anova was done to determine significance (n=6 both groups). B) 7860 WT (gray) and A4KO (red) were stained with CSFE and cultured for 48 hr. Representative histogram showing CSFE fluorescent intensity. C) Graph depics the average migration of the indicated CAKi-1 cells ± s.d. Welch’s t test was done to determine significance (n=6). D). The indicated CAKi-1 cells were grown in non-adherent conditions and the the number colonies were counted. Representative images of colonies formed in non-adherent conditions. E) Immunoblot for V5 in CAKi-1 A4KO cells mock transfected or transfected with full length ANGPTL4-V5 (FL) or N-terminus ANGPTL4-v5 (N-term). α-Tubulin is shown as a loading control. F) Immunoblot for V5 in the conditioned media (CM) of 786O A4KO cells mock transfected or transfected with nANGPTL4-V5. α-Tubulin the cell lysate (lys) is shown as a loading control.
