# Supplementary material for: ANGPTL4 Suppresses Clear Cell Renal Cell Carcinoma via Inhibition of Lysosomal Acid Lipase
Source: Cancer Res Commun. 2024 Aug 27;4(8):2242–54. doi: 10.1158/2767-9764.CRC-24-0016 (PMC11348483; doi:10.1158/2767-9764.CRC-24-0016)
Supplement: Supplementary Figure S4 [file crc-24-0016_supplementary_figure_s4_suppsf4.docx]

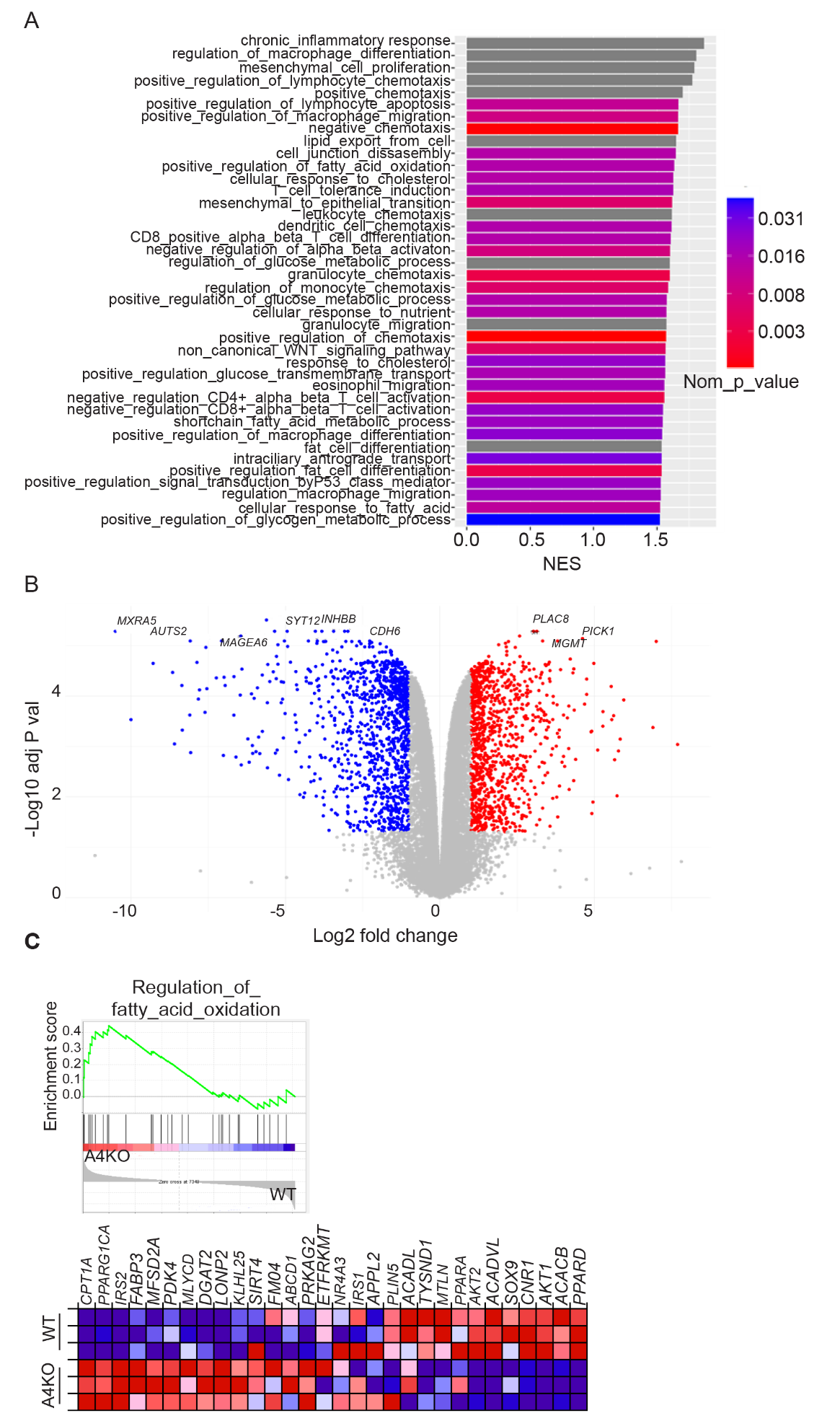


**Supplemental Figure S4.** A) GSEA was performed on RNAseq data of CAKi-1 A4KO and WT cells. Graph depicts the nominal enrichment score (NES) of immune and metabolism related gene sets that were enriched – had a FDR < 0.25 - in A4KO cells. The nominal P value is indicated by the heat bar. Grey bars had a nominal P value that was lower than the threshold (0.0001) for the package used and thus had a value of 0. B) Volcano plot for 786O A4KO versus WT cells. Genes with an absolute value log2 fold change ≥ 1 and an adjusted P value of ≤ 0.05 are indicated. Genes upregulated in A4 KO cells are red and genes downregulated in A4KO cells are blue. C) GSEA was performed on RNAseq data of 786O A4KO and WT cells. Upper panel: enrichment plot for the indicated gene set. Lower panel: heat map of the indicated gene set showing differential expression of the genes in the gene set in each group.
